# Association between causes of peritoneal dialysis technique failure and all-cause mortality

**DOI:** 10.1038/s41598-018-22335-4

**Published:** 2018-03-05

**Authors:** Jenny H. C. Chen, David W. Johnson, Carmel Hawley, Neil Boudville, Wai H. Lim

**Affiliations:** 1 Department of Nephrology, Prince of Wales Hospital, Sydney, Australia; 2 School of Medicine, University of New South, Sydney, Australia; 3 Department of Nephrology, University of Queensland at Princess Alexandra Hospital, Brisbane, Australia; 4 School of Medicine, University of Queensland, Brisbane, Australia; 5 Translational Research Institute, Brisbane, Australia; 6 Australasian Kidney Trials Network, Brisbane, Australia; 7 School of Medicine and Pharmacology, University of Western Australia, Perth, Australia; 8 Department of Renal Medicine, Sir Charles Gairdner Hospital, Perth, Australia

## Abstract

Technique failure is a frequent complication of peritoneal dialysis (PD), but the association between causes of death-censored technique failure and mortality remains unclear. Using Australian and New Zealand Dialysis and Transplant (ANZDATA) registry data, we examined the associations between technique failure causes and mortality in all incident PD patients who experienced technique failure between 1989–2014. Of 4663 patients, 2415 experienced technique failure attributed to infection, 883 to inadequate dialysis, 836 to mechanical failure and 529 to social reasons. Compared to infection, the adjusted hazard ratios (HR) for all-cause mortality in the first 2 years were 0.83 (95%CI 0.70–0.98) for inadequate dialysis, 0.78 (95%CI 0.66–0.93) for mechanical failure and 1.46 (95%CI 1.24–1.72) for social reasons. The estimates from the competing risk models were similar. There was an interaction between age and causes of technique failure (p_interaction_ < 0.001), such that the greatest premature mortality was observed in patients aged >60 years post social-related technique failure. There was no association between causes of technique failure and mortality beyond 2 years. In conclusion, infection and social-related technique failure are associated with premature mortality within 2 years post technique failure. Future studies examining the associations may help to improve outcomes in these patients.

## Introduction

Despite the increasing acceptance of peritoneal dialysis (PD) as a treatment for patients with end-stage kidney disease (ESKD), PD technique failure remains an important and frequent complication of PD. In Australia and New Zealand, almost 25% of prevalent PD patients experienced PD technique failure annually, with similar trends being observed in other countries including the United States and the United Kingdom^1–3^.

Peritonitis continues to be one of the most important risk factors for PD technique failure^4^, and contributes to almost 50% of death-censored PD technique failure in Australia, with similar observations being reported in other countries^5–7^. In an analysis of the Australian and New Zealand Dialysis and Transplant (ANZDATA) registry of 6639 incident PD patients, 10% of patients who had experienced peritonitis died within 30 days of peritonitis^8^, with similar findings corroborated in other studies^9^. These observations suggest that peritonitis is an important risk factor for both PD technique failure and premature mortality. We therefore hypothesized that PD patients who had experienced infection-related PD technique failure were more likely to die following PD technique failure compared to those who had experienced PD technique failure from other causes. The aims of this study were to examine the associations between causes of PD technique failure (other than death) and subsequent all-cause and cause-specific mortality, and to determine whether this association was modified by age.

## Results

### Baseline Characteristics

**Figure 1:**
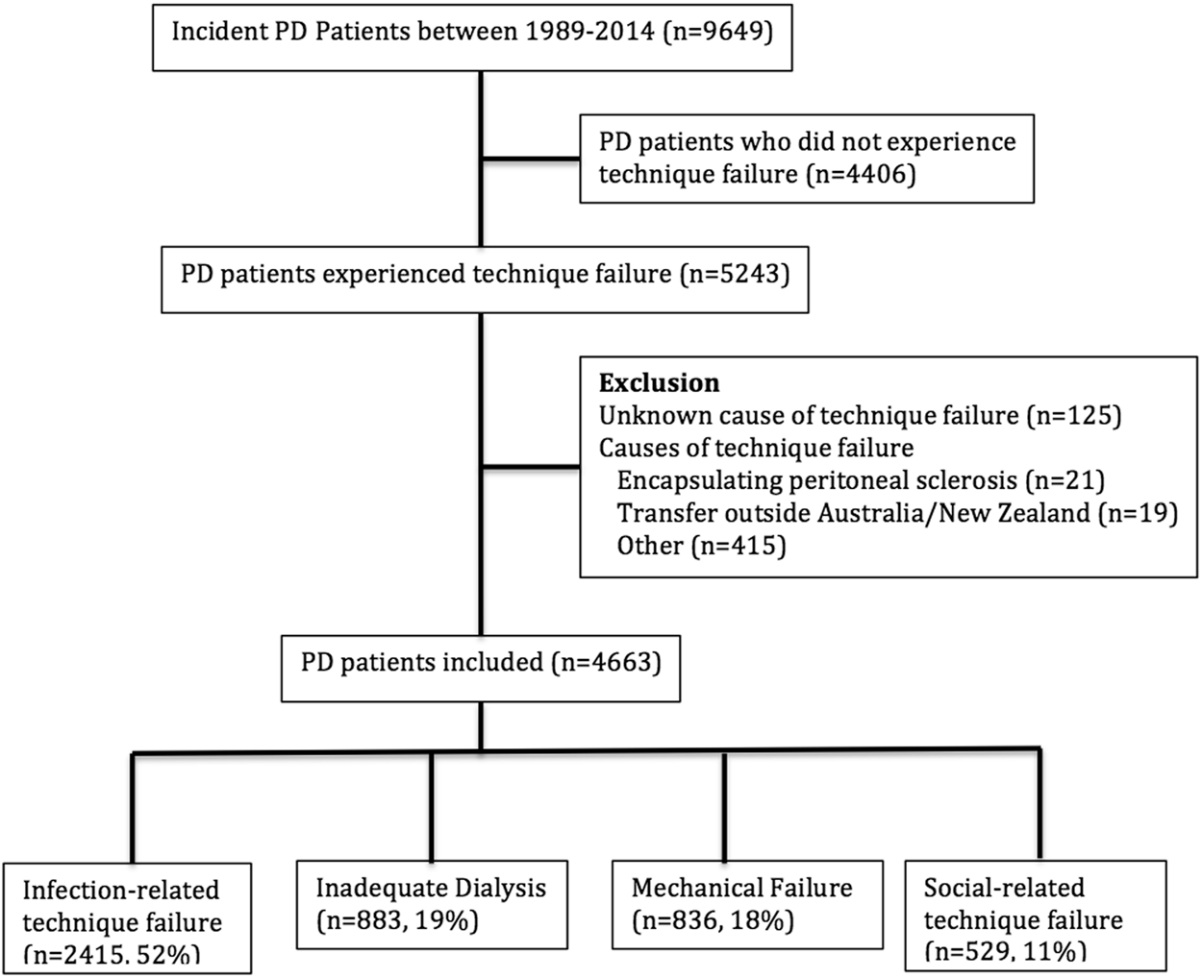
Patient flowchart for peritoneal dialysis patients who experienced peritoneal dialysis technique failure between 1989 and 2014.

Of 9649 incident PD patients between 1989 and 2014, 5243 (54%) experienced PD technique failure. Over 50% of the PD technique failure was attributed to infection (n = 2415, 52%), followed by inadequate dialysis (n = 883, 19%), mechanical failure (n = 836, 18%) and social reasons (n = 529, 11%) (Fig. 1). The median [IQR] follow-up time for patients who had experienced technique failure was 2.1 [3.4] years, with shorter follow-up duration for patients who had experienced social-related technique failure (1.7 [3.2] years), compared to patients who had experienced infection (2.2 [3.6] years), inadequate dialysis (2.1 [3.2] years) and mechanical-related (2.0 [3.2] years) causes of PD technique failure (p < 0.001). Table 1 shows the baseline characteristics stratified by causes of PD technique failure.

**Table 1:** Baseline characteristics of end-stage kidney disease patients who have experienced different causes of peritoneal dialysis technique failure (n = 4663).

	Infection (n = 2415)	Inadequate Dialysis (n = 883)	Mechanical (n = 836)	Social (n = 529)	Total (n = 4663)	p-value
Demographics						
Male (n, %)	1219 (51)	574 (65)	436 (52)	313 (59)	2542 (55)	<0.001
Age starting PD (year, mean ± SD)	59.0 ± 13.6	54.9 ± 14.6	59.3 ± 13.2	59.2 ± 16.1	58.3 ± 14.1	<0.001
Age of PD failure (year, mean ± SD)	60.8 ± 13.7	56.9 ± 14.7	60.5 ± 13.2	60.8 ± 16.3	60.0 ± 14.2	<0.001
BMI (mean ± SD)	26.8 ± 5.4	27.5 ± 5.4	27.0 ± 5.3	26.9 ± 5.2	27.0 ± 5.4	0.005
Ethnicity (n, %)						0.003
Caucasian	1629 (68)	639 (72)	608 (73)	481 (72)	3257 (70)	
Indigenous^	400 (17)	106 (12)	103 (12)	79 (15)	688 (15)	
Others	386 (16)	138 (16)	125 (15)	69 (13)	718 (15)	
Comorbidities						
Lung disease (n, %)	223 (9)	65 (7)	96 (12)	59 (11)	443 (10)	0.015
Coronary artery disease (n, %)	680 (28)	198 (22)	232 (28)	131 (25)	1241 (27)	0.006
Peripheral vascular disease (n, %)	373 (15)	134 (15)	124 (15)	99 (19)	730 (16)	0.22
Cerebrovascular disease (n, %)	255 (11)	62 (7)	96 (12)	64 (12)	477 (10)	0.003
Diabetes (n, %)	986 (41)	356 (40)	288 (34)	224 (42)	1854 (40)	0.006
Smoker (n, %)						0.13
Non-smoker	1109 (46)	444 (50)	384 (46)	230 (44)	2167 (47)	
Current smoker	338 (14)	125 (14)	113 (14)	87 (17)	663 (14)	
Ex-smoker	959 (40)	312 (35)	337 (40)	210 (40)	1818 (39)	
Cause of ESKD (n,%)						<0.001
Glomerulonephritis	560 (23)	248 (28)	204 (24)	113 (21)	1125 (24)	
Diabetes	821 (34)	288 (33)	213 (26)	175 (33)	1497 (32)	
Cystic	152 (6)	62 (7)	104 (12)	37 (7)	355 (8)	
Vascular	280 (12)	91 (10)	115 (14)	67 (13)	553 (12)	
Analgesic nephropathy	124 (5)	34 (4)	29 (4)	22 (4)	209 (5)	
Others	478 (20)	160 (18)	171 (21)	115 (22)	924 (20)	
Peritoneal dialysis characteristics						
Duration of PD (years, median ± IQR)	1.8 ± 2.2	2.0 ± 2.2	1.2 ± 1.7	1.6 ± 2.0	1.7 ± 2.1	<0.001
Initial peritoneal dialysis (n, %)						<0.001
CAPD	2024 (84)	703 (80)	682 (82)	397 (75)	3806 (82)	
APD	391 (16)	180 (20)	154 (18)	132 (25)	857 (18)	
Era						<0.001
1989–1998	472 (20)	107 (12)	113 (14)	78 (15)	770 (17)	
1999–2006	947 (39)	343 (39)	288 (34)	193 (37)	1771 (38)	
2007–2014	996 (41)	433 (49)	435 (52)	258 (49)	2122 (46)	
Proportion receiving kidney transplantation	427 (18)	224 (25)	185 (22)	82 (16)	918 (20)	<0.001

Data expressed as mean ± SD or as number (proportion). BMI – body mass index, PD – peritoneal dialysis, CAPD – continuous ambulatory peritoneal dialysis, APD – automated peritoneal dialysis, ESKD – end-stage kidney disease.

^^^Defined as Aboriginal and Torres Strait Islander, Maori and Pacific Islander.

A greater proportion of patients who had experienced infection-related technique failure were indigenous and were more likely to have diabetes as a comorbid condition compared to those who had experienced other causes of PD technique failure (17% vs. 13%, p = 0.001; 34% vs. 30%, p < 0.001, respectively). Compared to the included cohort, the characteristics of the patients who were excluded from this study (PD patients who had experienced PD technique failure from other causes) were younger (mean (SD) age of 55.0 (14.4) vs. 58.3 (14.1) years, p < 0.001), but the proportions with prevalent cardiovascular disease and diabetes were similar in both cohorts.

### Temporal trend in the causes of PD technique failure

The proportion of PD technique failure attributed to infection decreased over successive time periods (1989–1998: 61%; 1999–2006: 53%; 2007–2014: 47%, p < 0.001). In contrast, the proportion of PD technique failure attributed to inadequate dialysis and mechanical failure increased over successive eras (1989–1998: 14%, 15%; 1999–2006: 19%, 16%; 2007–2014: 20%, 20%, respectively; p < 0.001). The proportion of PD technique failure attributed to social reasons had remained unchanged (1989–1998: 10%, 1999–2006: 11%, and 2007–2014: 12%; p = 0.24).

### Mortality rates

Of the 5243 patients who had experienced PD technique failure, 3248 (62%) died. Mortality rates between 0–2 years were highest in patients who had experienced PD technique failure attributed to infection and social reasons. Beyond 2 years post-PD technique failure, mortality rates were similar among all groups (Table 2).

**Table 2:** Mortality following peritoneal dialysis technique failure.

	Infection (n = 2415)	Inadequate Dialysis (n = 883)	Mechanical (n = 836)	Social (n = 529)	Total (n = 4663)	p-value
All-cause mortality (per 100 patients, 95% CI)						
0–2 years (n = 4663)	29 (27–31)	21 (18–23)	20 (17–22)	35 (31–40)	26 (25–28)	<0.001
>2–5 years (n = 2379)	38 (36–41)	33 (29–38)	36 (32–41)	35 (29–42)	37 (35–39)	0.30
>5 years (n = 880)	64 (60–68)	62 (54–69)	58 (50–65)	59 (48–69)	62 (59–65)	0.48
Cardiac mortality (per 100 patients, 95% CI)						
0–2 years	10.3 (9.1–11.5)	7.9 (6.3–9.9)	6.8 (5.3–8.7)	10.8 (8.4–13.7)	9.3 (8.5–10.1)	0.007
>2–5 years	14.7 (12.8–16.7)	12.9 (10.1–16.3)	11.4 (8.7–14.8)	11.6 (8.0–16.4)	13.5 (12.1–14.9)	0.27
>5 years	23.3 (19.8–27.3)	26.1 (19.9–33.4)	23.8 (17.7–31.2)	19.3 (12.2–29.0)	23.5 (20.8–26.4)	0.70
Infectious mortality (per 100 patients, 95% CI)						
0–2 years	4.0 (3.3–4.8)	1.7 (1.0–2.8)	2.3 (1.5–3.5)	1.7 (0.9–3.2)	3.0 (2.5–3.5)	0.001
>2–5 years	4.8 (3.7–6.1)	2.0 (1.0–3.7)	4.4 (2.8–6.8)	3.1 (1.5–6.3)	4.0 (3.3–4.9)	0.059
>5 years	8.2 (6.1–11.0)	4.3 (2.1–8.7)	7.9 (4.6–13.4)	4.8 (1.9–11.7)	7.2 (5.6–9.1)	0.31
Withdrawal mortality (per 100 patients, 95% CI)						
0–2 years	7.4 (6.4–8.5)	4.5 (3.3–6.1)	4.2 (3.0–5.8)	13.6 (10.9–16.8)	7.0 (6.3–7.8)	<0.001
>2–5 years	9.6 (8.1–11.3)	8.7 (6.5–11.7)	10.4 (7.8–13.7)	10.2 (6.9–14.9)	9.6 (8.5–10.9)	0.85
>5 years	17.7 (14.6–21.4)	18.6 (13.4–25.4)	17.9 (12.6–24.8)	16.9 (10.3–26.3)	17.8 (15.5–20.5)	0.99

Data expressed as median ± IQR, as number (proportion) or as mortality rate per 100 patients (95% confidence intervals).

### Association between causes of PD technique failure and all-cause mortality

**Figure 2:**
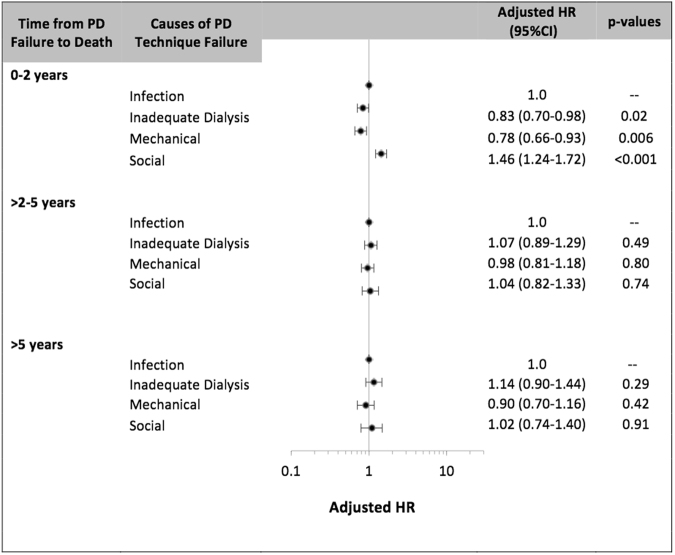
Association between causes of peritoneal dialysis technique failure and all-cause mortality post peritoneal dialysis technique failure*; the data is shown by the number of years following PD technique failure*.

Between 0 and 2 years post-PD technique failure, compared to patients who had experienced infection-related PD technique failure, the adjusted HR of all-cause mortality were 0.83 (95% CI 0.70–0.98), 0.78 (95% CI 0.66–0.93) and 1.46 (1.24–1.72), respectively for patients who had experienced inadequate dialysis, mechanical failure and social-related technique failure. Other covariates associated with mortality are shown in Table 3. There was no association between causes of PD technique failure and mortality beyond 2 years post-PD technique failure in the Cox regression models (Fig. 2).

**Table 3:** Association between causes of peritoneal dialysis technique failure and all-cause mortality between 0–2 years post PD technique failure.

	Multivariate (HR)	Competing Risk (SHR)
Causes of technique failure		
Infection	1.0	1.0
Inadequate dialysis	0.83 (0.70–0.98, 0.027)	0.83 (0.70–0.98, 0.03)
Mechanical	0.78 (0.66–0.93, 0.006)	0.78 (0.66–0.93, 0.006)
Social	1.46 (1.24–1.72, <0.001)	1.47 (1.25–1.73, < 0.001)
Age^#^	1.03 (1.03–1.04, <0.001)	1.04 (1.03–1.04, <0.001)
Gender		
Male	0.95 (0.84–1.08, 0.44)	0.94 (0.83–1.06, 0.33)
Race		
Caucasian	1.0	1.0
Indigenous*	0.95 (0.80–1.14, 0.60)	1.00 (0.83–1.20, 1.00)
Other	0.75 (0.63–0.90, 0.002)	0.76 (0.64–0.92, 0.004)
Comorbidities		
Lung disease	1.16 (0.97–1.38, 0.10)	1.17 (0.97–1.39, 0.09)
Coronary artery disease	1.13 (0.99–1.28, 0.07)	1.14 (1.00–1.30, 0.06)
Peripheral vascular disease	1.16 (1.00–1.35, 0.05)	1.17 (1.00–1.36, 0.05)
Cerebrovascular disease	1.23 (1.04–1.46, 0.02)	1.24 (1.04–1.48, 0.02)
Diabetes	1.26 (1.02–1.56, 0.03)	1.28 (1.03–1.59, 0.02)
Causes of ESKD		
Glomerulonephritis	1.0	1.0
Diabetic nephropathy	1.50 (1.17–1.92, 0.001)	1.53 (1.20–1.96, 0.001)
Cystic kidney disease	0.69 (0.50–0.95, 0.03)	0.68 (0.49–0.94, 0.02)
Renovascular disease	1.32 (1.07–1.62, 0.01)	1.34 (1.08–1.65, 0.007)
Analgesic nephropathy	1.31 (1.00–1.71, 0.05)	1.32 (1.00–1.73, 0.05)
Other	1.48 (1.23–1.77, <0.001)	1.49 (1.24–1.80, <0.001)
Smoking history		
Non-smoker	1.0	1.0
Current smoker	1.25 (1.04–1.50, 0.02)	1.29 (1.08–1.55, 0.006)
Ex-smoker	1.00 (0.88–1.14, 0.99)	1.01 (0.88–1.15, 0.92)
PD duration		
</=1 year	1.0	1.0
1–2 years	1.37 (1.19–1.58, <0.001)	1.32 (1.14–1.52, <0.001)
>2 years	2.25 (1.92–2.63, <0.001)	1.99 (1.70–2.33, <0.001)
PD modality		
CAPD	1.0	1.0
APD	0.99 (0.86–1.15, 0.93)	1.00 (0.86–1.16, 1.00)
Era		
1990–1998	1.0	1.0
1999–2006	0.75 (0.64–0.87, <0.001)	0.74 (0.63–0.86, <0.001)
2007–2014	0.56 (0.47–0.66, <0.001)	0.54 (0.45–0.63, <0.001)
BMI		
Normal (18.5–24.9)	1.0	1.0
Overweight (25.0–29.9)	0.76 (0.67–0.89, <0.001)	0.77 (0.68–0.89, <0.001)
Obese (>30.0)	0.80 (0.68–0.93, 0.005)	0.81 (0.69–0.95, 0.009)
Underweight (<18.5)	1.46 (1.07–1.98, 0.02)	1.48 (1.08–2.04, 0.02)

Data expressed as hazard ratio (95% confidence intervals, p-value). SHR- subdistribution hazard ratios. CAPD – continuous ambulatory peritoneal dialysis. APD – automated peritoneal dialysis.

#There was a significant interaction between age and causes of technique failure and the outcome of all cause mortality in the first 2 years post PD technique failure (p < 0.001).

**Figure 3:**
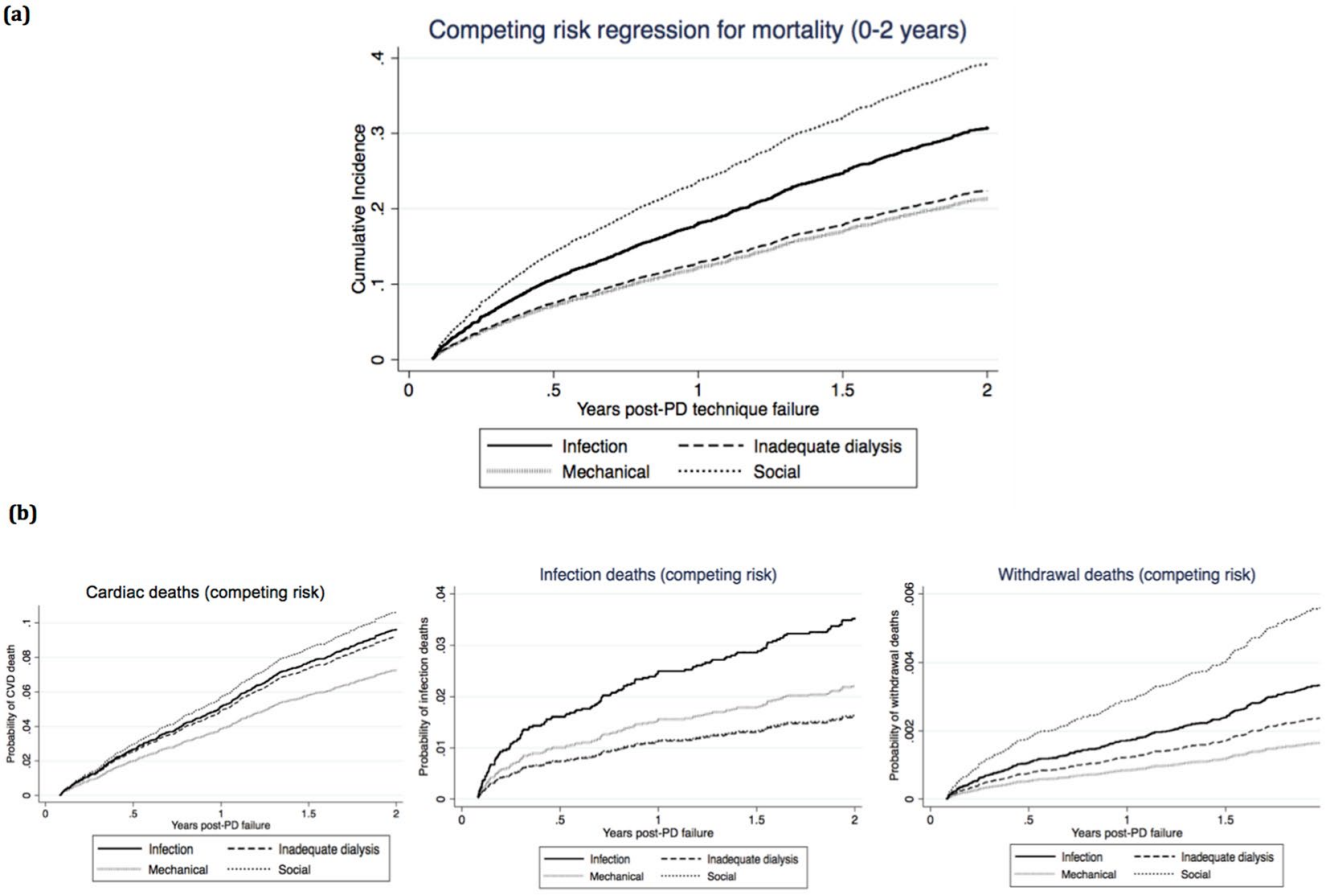
Competing risk analyses for mortality. (**a**) Competing risk regression for all-cause mortality 0–2 years post PD technique failure. (**b**) Competing risk regression for cause-specific mortality 0–2 years post PD technique failure.

In the competing risk analysis, compared to patients who had experienced infection-related PD technique failure, the adjusted subdistribution HR for mortality between 0 and 2 years post-PD technique failure in patients who had experienced inadequate dialysis, mechanical failure and social reasons were 0.83 (95% CI 0.70–0.98), 0.78 (95% CI 0.66–0.93) and 1.47 (95%CI 1.24–1.73), respectively (Fig. 3a). Other covariates in the competing risk models that were associated with mortality are shown in Table 3.

### Sensitivity analyses

In sensitivity analyses, similar association between causes of PD technique failure and all-cause morality was observed within the first six months and one year post technique failure, but not observed beyond the first 2 years. Between 2 and 3 years post-PD technique failure, compared to patients who had experienced infection-related PD technique failure, the adjusted HR of all-cause mortality were 1.04 (95% CI 0.78–1.39), 0.97 (95% CI 0.73–1.30) and 1.10 (0.77–1.56), respectively for patients who had experienced inadequate dialysis, mechanical failure and social-related technique failure.

### Cause-specific mortality

Cardiac death was the most common cause of death post-PD technique failure (n = 959, 36%), followed by dialysis withdrawal (n = 712, 27%) and infection (n = 297, 11%) (Supplementary Fig. S1). Between 0 and 2 years post-PD technique failure, the most common causes of death in patients who had experienced PD technique failure attributed to infection and social reasons were cardiovascular disease and withdrawal from dialysis, respectively. Beyond 2 years, cause-specific mortality rates were similar among all groups (Table 2).

**Figure 4:**
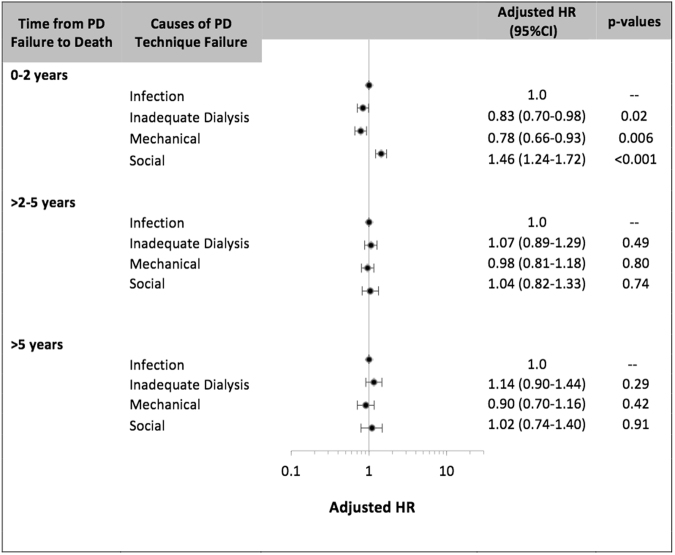
Association between causes of peritoneal dialysis technique failure and cause-specific mortality 0–2 years post PD technique failure.

Compared to patients who had experienced infection-related PD technique failure, the adjusted HR for cardiac mortality between 0 and 2 years post-PD technique failure were 0.92 (95% CI 0.70–1.20), 0.82 (95% CI 0.61–1.10), and 1.31 (95%CI 0.98–1.75) for patients who had inadequate dialysis, mechanical failure, and social-related failure, respectively. The adjusted HR for infection and withdrawal-related mortality are shown in Fig. 4. There was no association between causes of PD technique failure and cause-specific mortality beyond 2 years post-PD technique failure in the Cox regression models.

In the competing risk model, compared to patients who had experienced infection-related PD technique failure, the adjusted subdistribution HR for cardiac mortality within the first 2 years post-technique failure were 0.95 (95% CI 0.30–1.25), 0.75 (95%CI 0.56–1.00), 1.11 (95% CI 0.83–1.49) for patients who experienced inadequate dialysis, mechanical and social-related failure, respectively. The competing risk regression results for infection and withdrawal mortality are shown in Fig. 3b.

### Interaction between causes of PD technique failure and age for mortality

**Figure 5:**
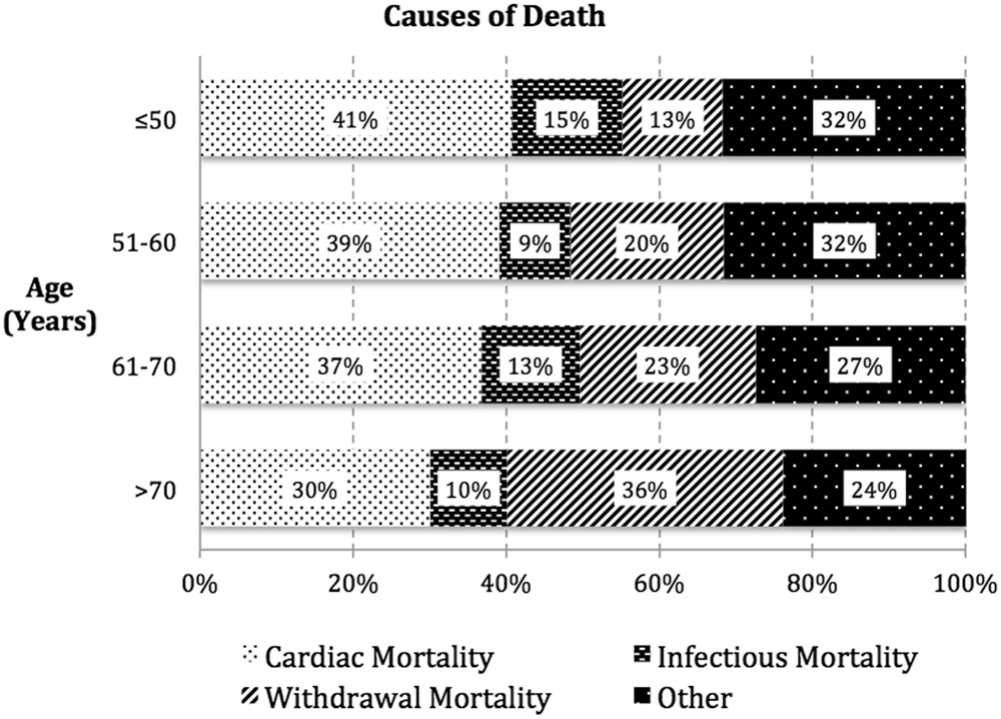
Proportions of cause-specific mortality 0–2 years post peritoneal dialysis technique failure stratified by age groups.

Patient age modified the association between causes of PD technique failure and all-cause mortality between 0 and 2 years post-PD technique failure (p-value for interaction <0.001), and therefore, age was categorized into quartiles (i.e. <= 50, 51–60, 61–70 and >70 years) for subsequent analysis. For patients aged >70 years (n = 1221), compared to infection-related PD technique failure, the adjusted HR for mortality within the first 2 years in patients who had experienced inadequate dialysis or mechanical failure (considered as a single group) was 0.78 (95% CI 0.62–0.97, p = 0.03), and 1.73 (95%CI 1.36–2.20, p < 0.001) for patients with social-related technique failure. Similar findings were observed in patients aged 61–70 years. For patients aged ≤50 years (n = 1126), compared to patients who had experienced infection-related technique failure, the adjusted HR for mortality within the first 2 years in patients who had experienced inadequate dialysis or mechanical failure was 0.79 (95% CI 0.54–1.16, p = 0.23), and 0.81 (95%CI 0.46–1.41, p = 0.45) for patients who had experienced social-related technique failure (Supplementary Fig. S2). Causes of death stratified by age groups between 0 and 2 years post-PD technique failure are shown in Fig. 5. Era did not modify the association between causes of PD technique failure and mortality at any time points.

## Discussion

This longitudinal cohort study over a period of two decades showed differential and time-dependent associations between causes of PD technique failure and all-cause mortality. Infection remained the most common cause of PD technique failure over successive eras, although the incidence was reduced by over 10% between 1989–1998 and 2007–2014. The greatest risk of mortality was observed in patients who had experienced social-related and infection-related PD technique failure, with 35% and 30% dying within the first 2 years post-PD technique failure, respectively. Cardiac mortality was the most frequent cause of death in patients who had experienced infection-related PD technique failure, whereas withdrawal mortality was the most frequent cause of death for social-related technique failure. Age modified the association between causes of PD technique failure and mortality such that, in older patients, only those who had experienced social-related technique failure were more likely to die within 2 years of PD technique failure, with the majority of the deaths attributed to psychosocial reasons. There were no associations between the other causes of PD technique failure and death in older patients.

Infection remained the most frequent cause of PD technique failure, with similar findings being observed in other countries across the United States, Canada, Europe and Asia^6,7,10–13^. Although a substantial reduction in the proportion of infection-related PD technique failure in Australia and New Zealand was observed over the last two decades, this was in direct contrast to observations in other countries. Data from the Canadian registry showed that infection-related technique failure had remained constant between 1995 and 2009, but PD technique failure attributed to inadequate PD had decreased over time^14^. Similarly, in Brazil, causes of technique failure did not change over time between 2005 and 2010^7^. Differences in study findings are likely to reflect dissimilar patient characteristics and systematic differences in the uptake/management of PD patients between countries as well as a lack of standardized definitions of PD technique failure attributed to infections or other causes.

Population cohort studies have consistently shown that PD patients who had experienced peritonitis were 2–6 times more likely to experience premature mortality within the first 1–2 months post-occurrence of peritonitis, with cardiac mortality being the predominant cause of mortality^8,9,15,16^. However, the association between different causes of PD technique failure and mortality remains unclear. In a single-centre study involving 286 incident PD patients between 2004–2010, risk of mortality doubled in patients who had experienced infection-related PD technique failure in the first 30 days post peritonitis, compared to other causes of PD technique failure^15^. In contrast, a cohort study from the United Kingdom showed that PD patients who had experienced social-related technique failure had greater mortality, compared to those who had experienced recurrent peritonitis or ultrafiltration-related PD technique failure (2-year survival 50%, 70%, 80%, respectively)^11^. The present study suggested that there was a time-dependent association between causes of PD technique failure and mortality, with infection and social-related PD technique failures having the highest risks of mortality within the first 2 years post-technique failure. However, there were no associations between causes of PD technique failure and mortality beyond 2 years post-technique failure. In patients with PD technique failure attributed to social reasons, 31% died from cardiac mortality and 35% died from withdrawal mortality, with 44% reported to have withdrawn for comorbid conditions such as cardiovascular disease and malignancy. The greater comorbid burden is likely to explain the higher mortality rates within the first few years post-PD technique failure in patients who had experienced social-related technique failure, as suggested by previous studies^17,18^. The finding of a higher risk of mortality in those who had experienced infection-related PD technique failure was predictable and consistent with previously published studies that have shown an independent association between infection and premature mortality. Even though patients who had experienced infection and social-related PD technique failures were more likely to die from infection and social-related mortality, respectively, cardiac death remained a common cause of death in these patients suggesting that causes of mortality were often multifactorial and intertwined. The lack of association between causes of PD technique failure and mortality beyond 2 years post-technique failure suggested a lack of persistent long-term adverse effects of PD failure attributed to infections or social causes.

A direct relationship between age and mortality risk has been consistently shown in incident PD patients^1,5,7,10,19^. In this study, we have shown that the association between PD technique failure and mortality was modified by age. Older patients with social-related PD technique failure were more likely to die prematurely post-technique failure compared to those who had experienced other causes of technique failures, likely related to the greater prevalence of comorbid burden in older patients. In contrast, younger patients aged between 51–60 years whom had experienced infection- or social-related PD technique failure appeared to have a higher risk of premature mortality (majority being cardiac death) compared to other causes of PD technique failure, suggesting that clinicians will need to be more vigilant in monitoring complications post-PD technique failure in this group of patients. Given the small number of events, we are unable to generate reliable estimates with any certainty.

There are several limitations that must be considered when interpreting the findings of this study. Even though there were multiple confounders adjusted for in the analysis, there are likely to be unmeasured and residual confounders. Data on the severity of comorbid conditions, medications, urine volume and volume status, socioeconomic status, care giver status, selection of ESKD patients for PD, management of PD and related complications, residual renal function at the time of PD technique failure, and other factors that could have potentially modified the association between causes of PD technique failures and mortality were not collected by the ANZDATA registry. In addition, the ANZDATA registry does not verify the reported causes of technique failure by the treating nephrologist and, as such, misclassification bias for the causes of PD technique failure is possible.

The association between causes of PD technique failure and mortality was time-dependent, with technique failure attributed to infection or social causes being associated with greater risks of mortality within the first 2-years following PD technique failure. Cardiac and infectious mortality were the commonest causes of premature mortality, particularly in younger patients who had experienced infection-related technique failure. Therefore, clinicians should be cognizant of the need for close follow-up with appropriate management of these patients. Future studies aiming to explore and understand these associations in greater detail may provide clinicians the opportunity to help better inform the development of individualized management plans in PD patients, which may help to improve their outcomes following transition to haemodialysis.

## Methods

### Study Population

All adult ESKD patients (aged ≥18 years) who had commenced PD between 1989 and 2014 and had experienced PD technique failure in Australia and New Zealand were included. PD technique failure was defined as transfer to haemodialysis for ≥30 days, and excluded technique failure attributed to death^12^. Patients who were maintained on PD for less than 90 days, those who were re-initiated on PD following technique failure and patients who had died whilst on maintenance PD were excluded. Data were censored as of 31^st^ December 2014. Approval of study by research ethics committee and informed consents were not required because only de-identified information was utilized for analysis. However, consents for inclusion in the ANZDATA registry were sought from all patients with end stage kidney disease in Australia and New Zealand.

### Data Collection

Baseline characteristics included gender, ethnicity, causes of primary ESKD, comorbidities at time of PD initiation (i.e. presence of diabetes, coronary artery disease, chronic lung disease and cerebrovascular disease), smoking history, body mass index (BMI), PD-related factors (such as PD duration and modality) and era (categorized as 1989–1998, 1999–2006 and 2007–2014).

### Exposure factors

Causes of PD technique failure were categorized into four groups (infection, inadequate dialysis, mechanical failure and social reasons)^12^. PD technique failures attributed to unknown or other causes were excluded from the study (n = 580; n = 125 unknown cause, n = 21 encapsulating peritoneal sclerosis, n = 19 transfer outside Australia or New Zealand, n = 415 other causes). Definitions of each causes of PD technique failure are shown in Supplementary Table S1.

### Clinical Outcomes

The primary outcome was all cause mortality. Secondary outcomes included cause-specific mortality (cardiac, infectious and dialysis withdrawal). Cardiac death included cardiac arrest, myocardial ischaemia and cardiac failure. Infection included septicaemia, peritonitis (bacterial, fungal, other) and infections at any sites. Dialysis withdrawal included psychosocial reasons, comorbidities and dialysis access difficulties (please see http://www.anzdata.org.au/forms/ANZDATA/anzdata_A3_2013.pdf).

### Statistical analysis

Data were expressed as numbers (percentages) for categorical data, means and standard deviations (SD) for normally distributed continuous data, and median and interquartile range [IQR] for continuous data that were not normally distributed, with comparisons between causes of PD technique failure using chi-square test, analysis of variance (ANOVA) and Kruskall-Wallis test, respectively where appropriate. The temporal trends in the causes of PD technique failure were examined across three time-periods of 1989–1998, 1999–2006 and 2007–2014. Mortality rates, expressed as the number of deaths per 100 patients, were calculated for each of the four causes of technique failure. The association between causes of PD technique failure and mortality was examined using adjusted Cox proportional hazard regression, censoring for kidney transplantation. The proportional hazard assumptions of the models were checked graphically by plotting the Schoenfeld residuals. As the models for mortality were found to violate the proportional hazards assumption, the time period between PD technique failure and mortality was separated into 0–2 years, >2–5 years and >5 years for analysis. Potential interaction between the exposure factor and age or era was examined using two-way interaction terms in the multivariable-adjusted model. Results were expressed as hazard ratios^13^ with 95% confidence intervals (95%CI). Covariates with p-values of <0.1 in the univariate models or with biological relevance (gender, smoking history and PD modality) were included in the multivariable-adjusted models. Sensitivity analyses for all-cause mortality were conducted for 0–6 months, 0–1 year and 2–3 years post PD technique failure.

### Competing risk analysis

Competing risk regression analyses were conducted for all-cause mortality (kidney transplantation considered as a competing event) and cause-specific mortality (cardiac, infection and withdrawal-related mortality) using the method described by Fine and Gray^20^. The stratified proportional subdistribution HR were calculated to estimate the exposure and covariate effects on the cumulative incidence function. Covariates included in the competing risk models were identical to those included in the Cox regression models. Statistical evaluation was performed using the SPSS statistical software program (version 24: SPSS, North Sydney, Australia) and STATA statistical software 9.4. P-values of less than 0.05 were considered statistically significant.

### Data Availability

The authors confirm that all data underlying the findings are fully available without restriction. The primary dataset for this manuscript was generated and made available to the authors by the Australian and New Zealand Dialysis and Transplant (ANZDATA) Registry, Adelaide, Australia. The ANZDATA Data Use Agreement between the ANZDATA Registry and the authors does not allow the authors to make the data publicly available. The authors confirm that all data underlying the findings can be obtained without restriction from the ANZDATA Registry. The interested researchers are advised to contact the ANZDATA Registry independently (email address requests@anzdata.org.au).

## Electronic supplementary material

Supplementary Materials
