# Supplementary material for: Association between causes of peritoneal dialysis technique failure and all-cause mortality
Source: Sci Rep. 2018 Mar 5;8:3980. doi: 10.1038/s41598-018-22335-4 (PMC5838094; doi:10.1038/s41598-018-22335-4)
Supplement: Supplementary file 1 — Supplementary Materials [file 41598_2018_22335_MOESM1_ESM.docx]

**Association between causes of peritoneal dialysis technique failure and all-cause mortality**

Jenny HC Chen, David W Johnson, Carmel Hawley, Neil Boudville, Wai H Lim

**Supplementary materials**

**Supplementary Table S1**: Causes of PD technique failure

| Causes of PD Technique Failure |
| --- |
| Infection-Related PD Failure  Peritonitis  Tunnel/exit site infection  Diverticulitis and abdominal abscess |
| Inadequate Dialysis PD Failure  Inadequate solute clearance  Inadequate fluid ultrafiltration  Excessive fluid ultrafiltration  Malnutrition |
| Mechanical PD Failure  Dialysate leak  Surgery  Hernia  Abdominal pain  Catheter complications  Haemoperitoneum |
| Social-Related PD Failure  Inability to manage self-care  Patient preference  Geographical access |

**Supplementary Figure S1:** Causes of death post peritoneal dialysis technique failure.

**Supplementary Figure S2**: Association between causes of PD technique failure and all-cause mortality 0-2 years post PD technique failure stratified by age groups.

| **Age** | **Causes of PD**  **Technique Failure** |  | **Adjusted HR (95% CI)** | **p-value** |
| --- | --- | --- | --- | --- |
|  |  | 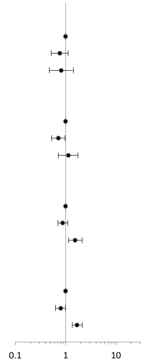   \| **Adjusted HR** \| \| --- \| |  |  |
|  | |  |  |  |
| **≤50 years** (n=1126) | |  |  |  |
|  | Infection |  | 1.0 | -- |
|  | Inadequate Dialysis/Mechanical |  | 0.79 (0.54-1.16) | 0.23 |
|  | Social |  | 0.81 (0.46-1.41) | 0.45 |
|  |  |  |  |  |
|  |  |  |  |  |
| **51-60 years** (n=1028) | |  |  |  |
|  | Infection |  | 1.0 | -- |
|  | Inadequate Dialysis/Mechanical |  | 0.71 (0.52-0.96) | 0.03 |
|  | Social |  | 1.21 (0.77-1.90) | 0.41 |
|  |  |  |  |  |
|  |  |  |  |  |
| **61-70 years** (n=1288) | |  |  |  |
|  | Infection |  | 1.0 | -- |
|  | Inadequate Dialysis/Mechanical |  | 0.87 (0.70-1.10) | 0.25 |
|  | Social |  | 1.51 (1.09-2.08) | 0.01 |
|  |  |  |  |  |
|  |  |  |  |  |
| **>70 years** (n=1221) | |  |  |  |
|  | Infection |  | 1.0 | -- |
|  | Inadequate Dialysis/Mechanical |  | 0.78 (0.62-0.97) | 0.03 |
|  | Social |  | 1.73 (1.36-2.20) | <0.001 |
|  |  |  |  |  |
|  |  |  |  |  |
|  |  |  |  |  |
|  |  |  |  |  |
